# Permanent Hydrophobic Surface Treatment Combined with Solvent Vapor-Assisted Thermal Bonding for Mass Production of Cyclic Olefin Copolymer Microfluidic Chips

**DOI:** 10.1021/acsomega.2c01948

**Published:** 2022-05-31

**Authors:** Tianyu Guan, Sineenat Yuket, Hengji Cong, Douglas William Carton, Nan Zhang

**Affiliations:** † Centre of Micro/Nano Manufacturing Technology (MNMT-Dublin), School of Mechanical & Materials Engineering, University College Dublin, Dublin 4 Dublin, Ireland; ‡ MiNAN Technologies, NovaUCD, Belfield, Dublin 4 Dublin, Ireland

## Abstract

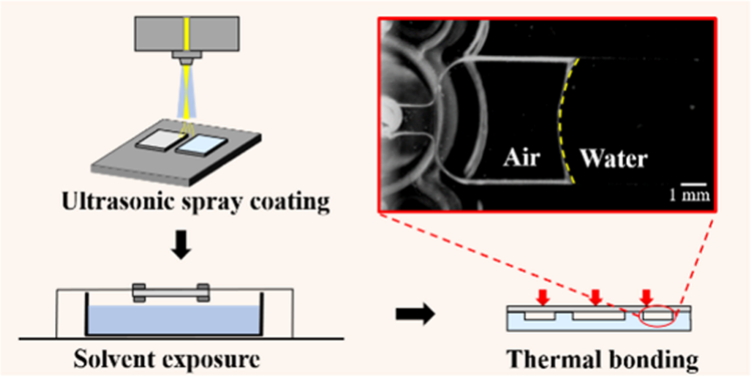

A hydrophobic surface modification followed by solvent vapor-assisted thermal bonding was developed for the fabrication of cyclic olefin copolymer (COC) microfluidic chips. The modifier species 1*H*,1*H*,2*H*,2*H*-perfluorooctyl trichlorosilane (FOTS) was used to achieve the entrapment functionalization on the COC surface, and a hydrophobic surface was developed through the formation of a Si–O–Si crosslink network. The COC surface coated with 40 vol % cyclohexane, 59 vol % acetone, and 1 vol % FOTS by ultrasonic spray 10 and 20 times maintained its hydrophobicity with the water contact angle increasing from ∼86 to ∼115° after storage for 3 weeks. The solvent vapor-assisted thermal bonding was optimized to achieve high bond strength and good channel integrity. The results revealed that the COC chips exposed to 60 vol % cyclohexane and 40 vol % acetone for 120 s have the highest bond strength, with a burst pressure of ∼17 bar, which is sufficient for microfluidics applications such as droplet generation. After bonding, the channel maintained its integrity without any channel collapse. The hydrophobicity was also maintained, proved by the water contact angle of ∼115° on the bonded film, as well as the curved shape of water flow in the chip channel by capillary test. The combined hydrophobic treatment and solvent bonding process show significant benefits for scale-up production compared to conventional hydrophilic treatment for bonding and hydrophobic treatment using surface grafting or chemical vapor deposition since it does not require nasty chemistry, long-term treatment, vacuum chamber, and can be integrated into production line easily. Such a process can also be extended to permanent hydrophilic treatment combined with the bonding process and will lay a foundation for low-cost mass production of plastic microfluidic cartridges.

## 1 Introduction

In recent years, microfluidics with hydrophobic surfaces have attracted much interest and have been widely used in diverse applications, including the modulation of protein adsorption and cell adhesion,^1^ the reduction of flow resistance in microfluidic chip channels,^2^ and the water-in-oil droplets generation in droplet-based microfluidics.^3^ The demand for hydrophobic microfluidics necessitates the fabrication, bonding, and surface modification of thermoplastic devices.^4^ Thermoplastic polymers, such as cyclic olefin copolymer (COC), cyclic olefin polymer (COP), poly(methyl methacrylate) (PMMA), and polycarbonate (PC), have been widely investigated as the substrates for microfluidic devices,^5^ due to their precise replication of micropatterns with high-quality surfaces. They are also suitable for mass production through injection molding and hot embossing at a low cost. Compared with other thermoplastics, COC has apparent advantages, such as good optical transparency, low water absorption, low autofluorescence, high chemical resistance, and good thermal resistance.^6^ However, native COC cannot provide sufficient hydrophobicity in some applications, including the formation of water-in-oil droplets, which requires a hydrophobic surface treatment on COC substrate. Nevertheless, the hydrophobic surface treatment will lead to a lack of wettability between two mating surfaces during bonding, hindering the sealing of COC microfluidics.

Bonding and hydrophobic surface treatment are two essential processes for fabricating microfluidic devices with hydrophobic surfaces. A wide variety of bonding techniques have been investigated for thermoplastic microfluidics, such as adhesive bonding, thermal fusion bonding, and solvent bonding. Adhesive bonding is simple to operate for sealing thermoplastic microfluidics. Liquid adhesives are typically applied on the chip surface, which could be cured after ultraviolet (UV) exposure or solvent evaporation.^7^ However, this method may cause channel clogging, which requires the removal of uncrosslinked adhesive trapped in the channel by organic solvent,^8^ making it difficult to carry out on a large scale. In thermal fusion bonding, thermoplastic chips are heated to a temperature around/above their glass-transition temperature (*T*_g_) and compressed by a hold pressure. This bonding method often results in higher bond strength as the complete diffusion of polymer chains occurs between two mating surfaces. However, microchannels are prone to collapse once applied with high temperature and pressure, making it challenging to maintain channel integrity.^9^ Meanwhile, to reduce the surface energy of bonding surfaces, UV/ozone or oxygen plasma treatment is typically applied to make surfaces more hydrophilic,^10^ resulting in a processing time often longer than 1 h.^7^ During solvent bonding, thermoplastic substrates are dissolved in organic solvents with similar solubility.^11^ After the polymer surfaces are solvated, the polymer chains are mobile and can easily diffuse across the solvation layer, forming an entanglement layer and resulting in a high bond strength.^12^ However, the immersion of polymer into the solvent is not an easily controllable process: the excessive solvent absorption in the polymer substrates could cause severe channel deformation once being mated under pressure.^7^

Various surface treatment and modification methods have been reported to obtain a highly hydrophobic surface of thermoplastics, including plasma treatment, graft polymerization, and entrapment functionalization. Ghosh et al. exposed the thermoplastic chips to the mixture of CF_4_ and O_2_ for plasma treatment to have a hydrophobic surface.^13^ However, this treatment is expensive and difficult to scale up. Meanwhile, treating the thin surface layer without changing the bulk properties of the thermoplastics is also a challenge for plasma treatment.^14^ Industrially, plasma-enhanced chemical vapor deposition is used to deposit hydrophobic polymers into the microchannels. However, due to the smaller channel size, such coating requires a longer diffusion time and is not so reliable due to restricted diffusion. Robust coatings can be produced by graft polymerization, with tunable chemical properties and precise control of local definitions.^6^ This surface modification typically involves two steps: surface activation and graft polymerization.^15^ Due to the lack of chemically reactive functional groups on thermoplastic surfaces, the activation process through UV treatment, plasma treatment, or high-energy electrons is required to generate reactive sites for further grafting processes. Then, the functional molecules with reactive groups will be covalently coupled to the surface.^15^ The whole operation process is relatively time-consuming and complex, making the modification process slow. During entrapment functionalization, the surface of thermoplastic is immersed in a solvent containing modifier species and the polymer surface will swell due to the interaction with the solvent. The polymer chains become mobile and entangled with modifier species during surface swelling. Then, the polymer surfaces deswell in the water moisture and the modifier species are firmly embedded on the polymer surface with the evaporation of solvent, and the surface properties are modified accordingly.

In most studies, bonding and hydrophobic treatment are conducted sequentially, as the surface energy required for bonding and surface treatment is the opposite. For the bonding process, the surface energy should be increased to improve the wettability and adhesion of two mating surfaces. In contrast, for hydrophobic surface treatment, the surface energy is decreased to reduce the wettability of microchannels. Therefore, modifying the channel wall to hydrophobicity after bonding becomes a challenging and time-consuming task due to this process sequence. It is difficult to quickly achieve the microfluidics bonding and hydrophobic surface treatment for scalable production. Su et al. prepared the solvent mixture with 1.0 vol % 1*H*,1*H*,2*H*,2*H*-perfluorooctyl trichlorosilane (FOTS) as a surface modifier, acetone and *n*-pentane as solvent bonding solution to bond and modify the hydrophilic paper chromatography (PC) chips to hydrophobicity in one step, and the bond strength was 3.8 MPa with the water contact angle of ∼117.8° on the modified and bonded PC substrate.^16^ However, they did not identify the scalability for applying the hydrophobic modifier. In all, laboratory treatment using surface grafting and other techniques involves nasty chemistry and takes a much longer time. Industrial surface treatment using plasma-enhanced chemical vapor deposition requires vacuum and is difficult for reliable treatment for tiny channels. It is still a significant challenge to develop a scalable, low-cost, and reliable process for the integration of microfluidic chips by combining surface treatment and bonding process.

In this work, a hydrophobic surface modification by ultrasonic spray coating on COC 8007 substrates followed by solvent vapor-assisted thermal bonding was developed to fabricate microfluidic chips. This combined method offers a cost-effective alternative for the large-scale production of chip assemblies with highly hydrophobic surfaces. Moreover, the surface treatment and bonding method were optimized to achieve a long-term stable hydrophobicity and high bond strength of the bonded COC chips. Each treatment was analyzed in terms of water contact angle, attenuated total reflection Fourier transform infrared (ATR-FTIR) spectra, and surface roughness to achieve the optimum surface treatment condition for high hydrophobicity and good optical clarity. After bonding, a leakage test followed by a burst pressure test was carried out to identify the bonding parameters that could achieve the highest bond strength. The channel integrity was also analyzed, and the stability of surface hydrophobicity was further proved by capillary test and water contact angle measurement after bonding. This work will lay a foundation for large-scale accessible surface treatment and bonding processes for other thermoplastics in microfluidic applications.

## 2 Material and Methods

### 2.1 Materials

Cyclic olefin copolymer (COC) pellets (Grade 8007) for chip fabrication and films (Grade 8007, 150 μm) were obtained from Topas (TOPAS Advanced polymers GmbH, Frankfurt, Germany). Cyclohexane (≥99.9%) and acetone (≥99.9%) were purchased from Sigma-Aldrich. 1*H*,1*H*,2*H*,2*H*-Perfluorooctyl trichlorosilane (FOTS; 97%) was obtained from Fischer Scientific. All reagents were used as received.

### 2.2 Microfabrication

**Figure 1:**
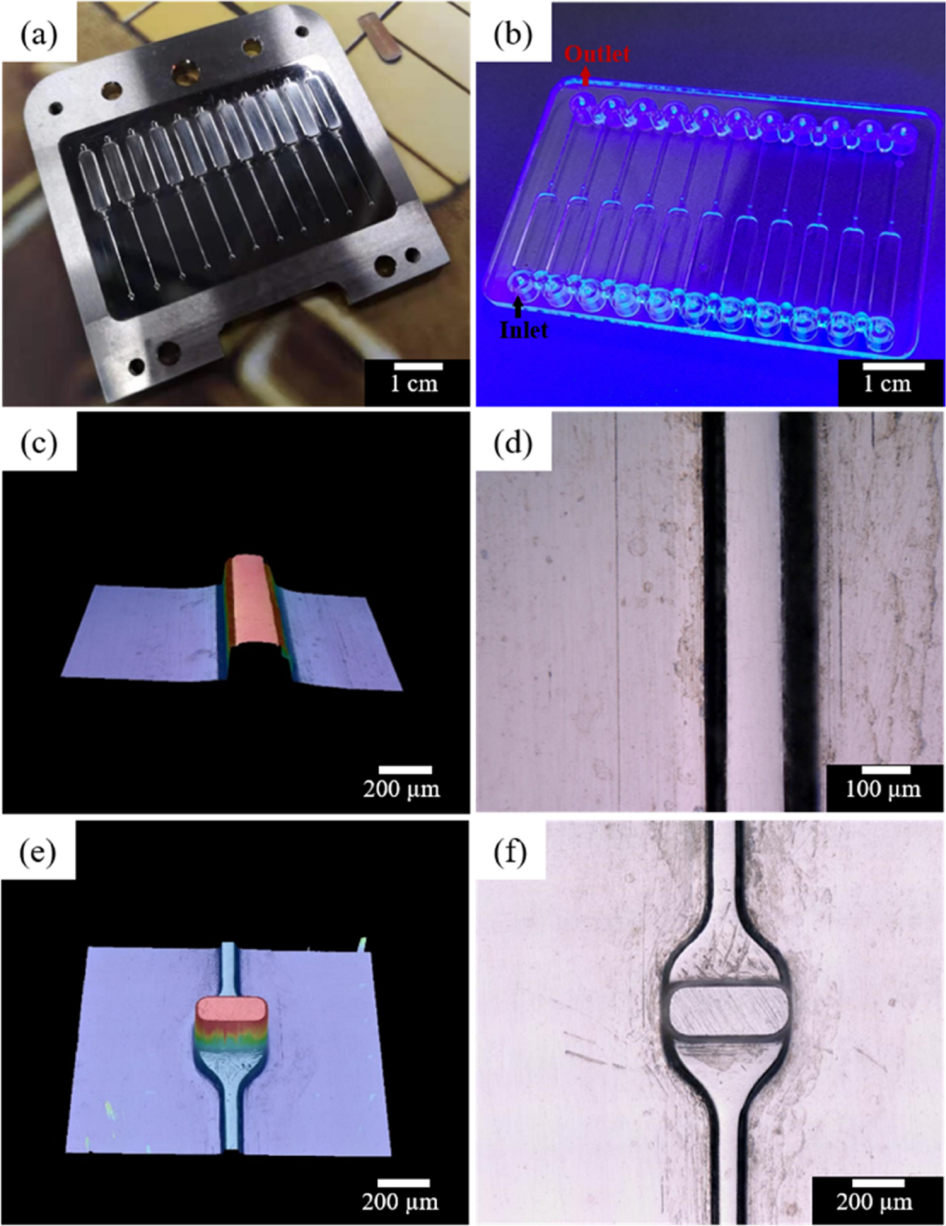
Optical image of high-precision tool steel mold insert (a) and injection-molded chip with micropatterns (b). (c–f) 3D and two-dimensional (2D) images of micropatterns on the mold.

The COC microfluidic chips with micropatterns were fabricated using an injection molding machine (FANUC ROBOSHOT S-2000i15B) with a high-precision tool steel mold (shown in Figure 1), and the mold insert was characterized by a three-dimensional (3D) microscope (Keyence VHX-5000). The COC-8007 pellets (glass-transition temperature 78 °C) were used as raw materials. The dimensions of the chips were 60 mm (length) × 40 mm (width) × 1.70 mm (height). The microfluidic channels on the chips were ∼100 μm deep and ∼100 μm wide at the smallest section. The mold temperature was set at 80 °C, and the nozzle temperature was 230 °C. The injection velocity was set as 100 mm/s with a shot size of 43 mm, and the holding pressure was 62 MPa for 5 s. The injection-molded COC chips had uniform surfaces without any defects. After injection molding, both COC chips and prepared COC films were washed with isopropanol in an ultrasonic bath for 15 min and dried with compressed air before bonding.

### 2.3 Surface Modification and Bonding

**Figure 2:**
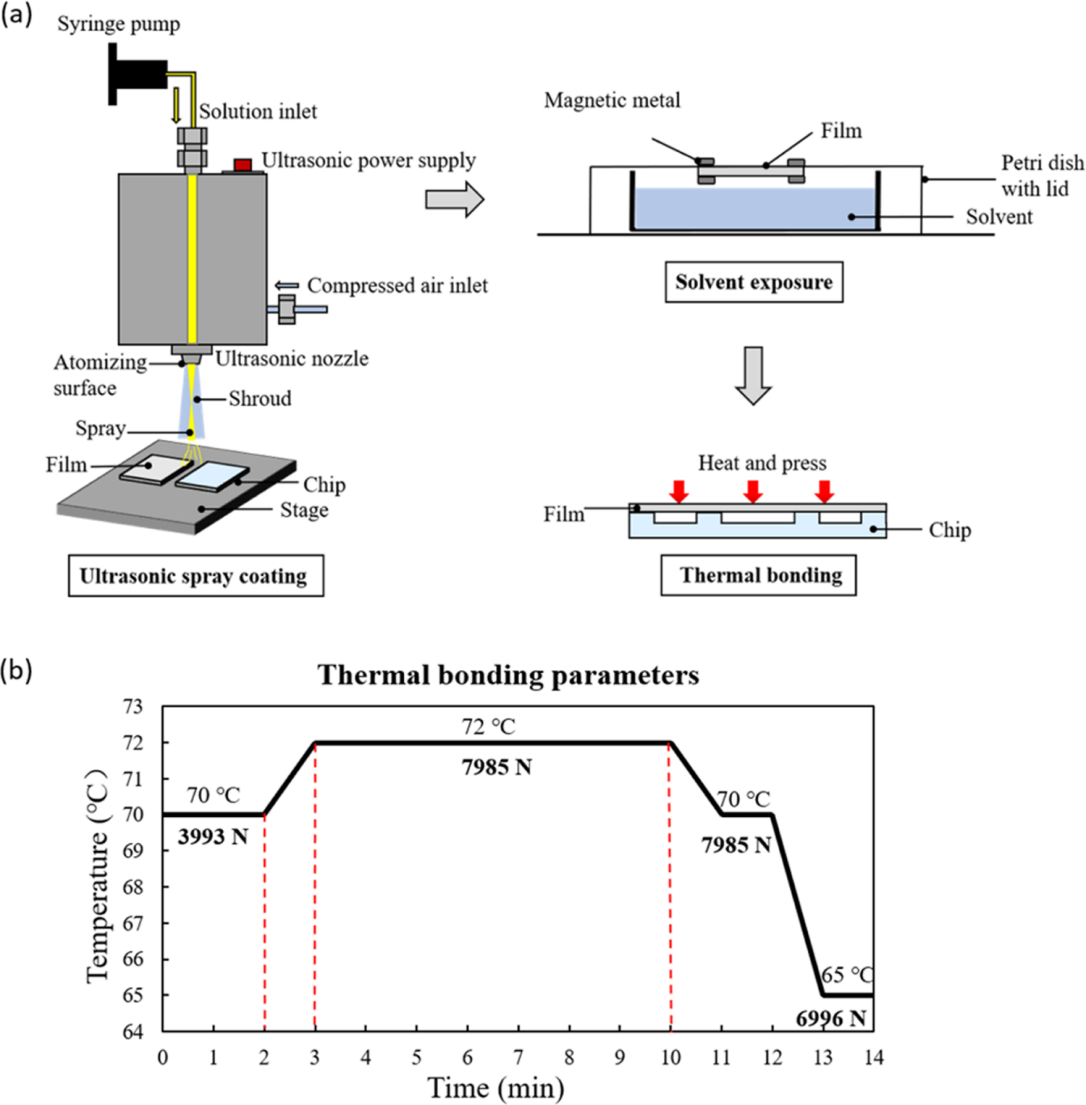
Schematic diagram of ultrasonic spray coating and subsequent solvent vapor-assisted thermal bonding process (a) and thermal bonding parameters (b).

The mixture solution of different concentrations of FOTS, cyclohexane, and acetone was used to modify the surface of both chips and films by ultrasonic spray coating for different coating times (as shown in Table 1 and Figure 2). The spray coating speed was 0.5 mL/min with an air pressure of 0.3 MPa and an ultrasonic current of 0.05 A. For each round of coating, there was 0.02 mL of the solution deposited on the chip surface, which contained 1% of FOTS. In our previous study, various concentrations of FOTS have been tried for surface treatment. However, the lower concentration could not achieve desired hydrophobicity, while the higher concentration would not allow bonding or gave a low bonding strength. Therefore, 1% FOTS was selected as the optimized concentration for surface treatment. In this study, 0.2 μL of FOTS were deposited onto the surface per round. For each sample, coatings were performed 5, 10, and 20 times.

**Table 1:** Surface Modification Parameters

vol % cyclohexane	vol % acetone	vol % FOTS	coating times for each sample
20	79	1	5, 10, 20
30	69	1	5, 10, 20
40	59	1	5, 10, 20

Subsequently, the modified films were exposed to the vapor of the prepared solvent mixture of 60% cyclohexane (30 mL) and 40% acetone (20 mL) for 60 and 120 s in a glass Petri dish with a lid (shown in Figure 2). The treated film was attached to the Petri dish lid by magnetic metals, and the distance between film and solvent liquid level was fixed as 1 cm. After exposure, the film cover was aligned in parallel with the modified chip in the holder in the hot embossing machine. The temperature and holding pressure were applied to the film and chip. As shown in Figure 2b, the bonding temperature was set at 72 °C, which is below the *T*_g_ of COC 8007 (78 °C), to maintain the integrity of the microstructure of the chip. Finally, a beaker of water (∼20 mL) was placed in an oven with the modified chips overnight to let the solvent evaporate in the atmospheric environment at room temperature. For the bonding process mentioned before, the unmodified COC film and chip were also bonded under the same solvent vapor bonding conditions and were characterized to compare the effect of surface modification on subsequent bonding.

### 2.4 Surface Analysis

To prove the performance of hydrophobic treatment, the modified chips were evaporated under the same condition as mentioned in Section 2.3 before the surface characterization. Water contact angles were measured on the surfaces of modified and unmodified chips using a contact angle goniometer (Ossila) at five different positions on each chip at five different time points (from before treatments to 10 days after the treatments). The ultrapure water was used for contact angle measurement, and the droplet volume was controlled at 10 μL by a pipette. Attenuated total reflection Fourier transform infrared (ATR-FTIR) spectroscopy was used to investigate the mechanisms of the surface modification on the COC surface. The surface roughness change of the COC chip before and after the modification was detected by an optical 3D profilometer (NPFlex). The modified film cover was placed on a pattern to compare the optical clarity with and without the hydrophobic surface treatment. Finally, to investigate the effect of coating times on the optical transmission properties of the COC film, the transmission of the native and coated COC film was carried out using an ultraviolet–visible (UV–vis) spectrophotometer (Agilent Technologies Ireland Ltd., Dublin, Ireland) in the range of 200–800 nm.

### 2.5 Bond Strength Analysis

#### 2.5.1 Leakage and Burst Test

A leakage test was performed before bonding characterization to ensure the films, and the chips were bonded successfully without any leakage. A drill was used to punch inlet and outlet holes on the chip side, and those holes enabled the fluid infusion in the microchannels. Silicone tubes were inserted into the inlets, and the blue ink solution was injected through the tubes into the microchannels by a syringe pump with a flow rate of 10 mL/min for better visualization of the leakage test.

The burst pressure test of the bonded chips was performed in four different channels to evaluate the bonding strength. During the test, the outlet hole of the channel was blocked by a plug. The burst pressure was tested by pumping water into the inlet hole of the channel with a manual test pump (EGA MASTER Manual Test Pump 60005) and by monitoring the pressure at which the bonded chips were disassembled.

#### 2.5.2 Channel Integrity Characterization

The channel integrity of the bonded chip was then investigated by the cross-sectional analysis under the microscope (Am scope led-144s). The chip was cut by saw, ground, and polished using P320, P600, P2500, and P4000 sandpaper with cooling water for smooth surface finishing before observation. The cross section of the coated and bonded chip was compared with the COC chip without treatment and bonding.

#### 2.5.3 Water Contact Angle Measurement and Capillary Effect Test

After bonding, the water contact angle was measured on the excess film cover to detect the effect of bonding on the hydrophobicity of the film. The capillary effect was performed to observe the flow shape affected by the surface modification and chip bonding. The deionized (DI) water was injected into the channel slowly until the injection flow was stopped at a certain point inside the channel to observe the interface between air and water. The flow shape at the interface was observed under the microscope (Am scope led-144s) to show the capillary effect inside the chip channel with and without hydrophobic modification.

## 3 Results and Discussion

### 3.1 Mechanism of Surface Modification and Bonding

#### 3.1.1 Mechanism of Solvent Vapor-Assisted Thermal Bonding

**Figure 3:**
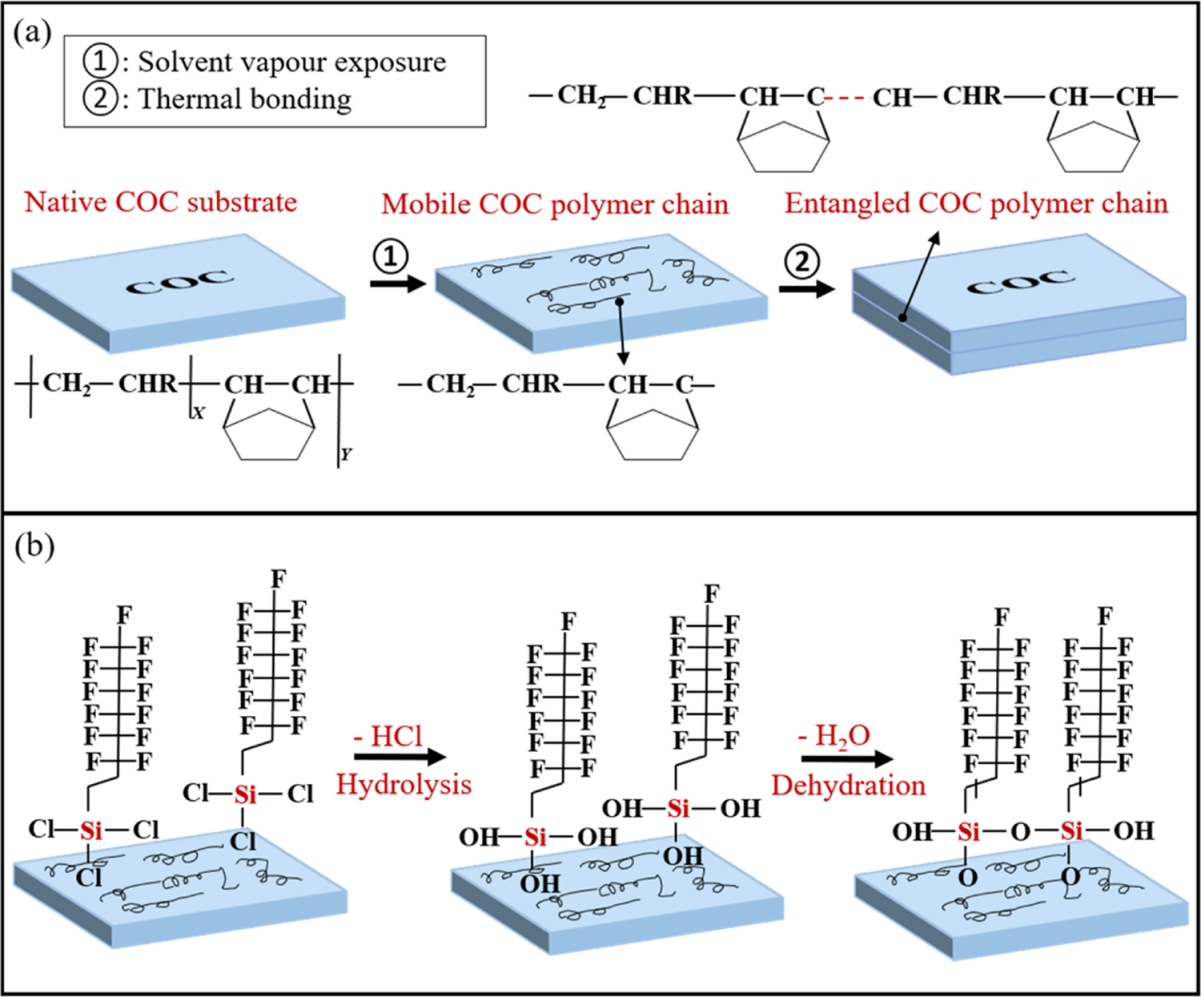
Mechanism of (a) solvent vapor-assisted thermal bonding and (b) hydrophobic surface modification by entrapment functionalization.

The solvent vapor-assisted thermal bonding was achieved using the combination of cyclohexane and acetone in the solution. It is possible that acetone acted as a sacrificial solvent while cyclohexane acted as a solvating solvent, which could be explained by the following reasons: first, COC has similar solubility with nonpolar organic solvents, such as hydrocarbons; the solubility parameter (δ) of COC is 17.7 [(J/cm^3^)^1/2^], and the δ of cyclohexane is 16.7 [(J/cm^3^)^1/2^], while the δ of acetone is 20.4 [(J/cm^3^)^1/2^].^12^ Therefore, COC prefers to dissolve in cyclohexane, and it is almost insoluble in polar organic solvent acetone.^7^ Second, acetone has a boiling point of 56.10 °C, while that of cyclohexane is 80.75 °C. After solvent vapor exposure, the COC cover film and substrate were aligned in the preheated thermal press machine.^17^ Once heated, acetone evaporated faster than cyclohexane, increasing the concentration of cyclohexane. After the COC chip and film were thermally pressed, all of the solvent evaporated, causing the mobilized COC polymer chains to entangle with each other on the film cover and chip substrate, leading to a strong bonding (shown in Figure 3a).^11^

#### 3.1.2 Mechanism of Hydrophobic Surface Modification through Entrapment Functionalization

During the solvent vapor-assisted thermal bonding process, the hydrophobic COC surface transformed into a more hydrophobic state. As shown in Figure 3b, during the surface treatment process, the modifier FOTS was immobilized on the COC surface.^16^ After the COC film cover and chip substrate were fed into the thermal press machine, the concentration of cyclohexane temporarily increased due to the higher evaporation rate of acetone, causing COC polymer chains to become mobile in the solvent vapor. When the COC surface material was swelling, it allowed the FOTS molecules to be embedded into its surface and entangled with these modifier molecules. After solvents were evaporated, these FOTS molecules were firmly embedded on the COC surface. The next step was the deswelling by water in the air so that the FOTS species could be fixed on the COC surface.^18^ During the deswelling process, the Si–Cl groups of the FOTS modifier would be gradually hydrolyzed by water in the air to become Si–OH groups.^19^ The Si–OH groups of adjacent FOTS molecules dehydrated spontaneously with each other, forming a Si–O–Si crosslinked network, which could further fix the FOTS molecules on the COC surface.^20^

### 3.2 Surface Treatment Optimization and Characterization

#### 3.2.1 Water Contact Angle Measurement

**Figure 4:**
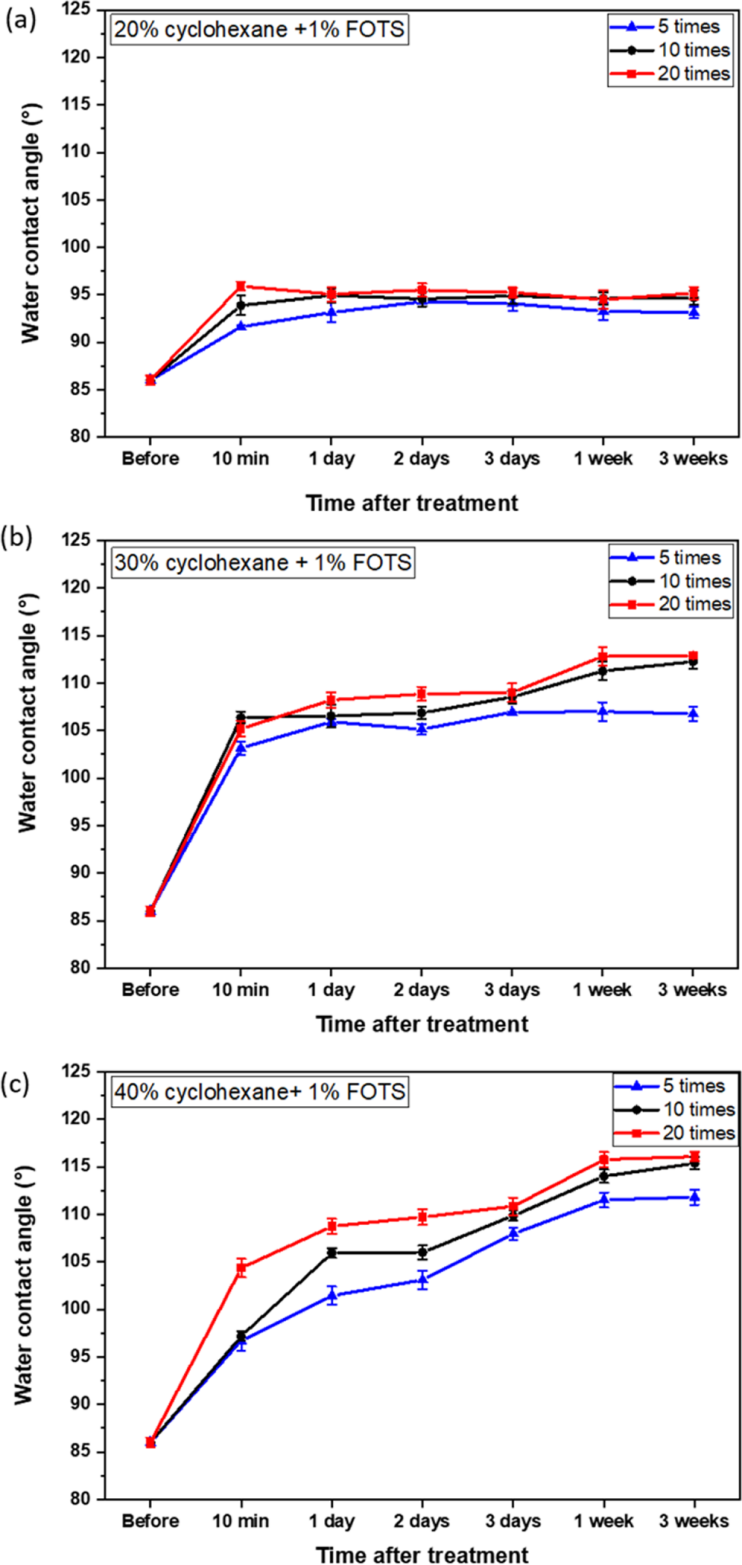
Changes in water contact angle on COC surface over time, after the surface treatment with (a) 20%, (b) 30%, and (c) 40% cyclohexane and 1% FOTS with different coating times.

The relationship between COC substrates’ hydrophobicity and cyclohexane concentration, coating times was investigated. The wettability of the COC surface was determined by measuring the water contact angle (WCA) of its surface. The hydrophobic surface plays an essential role in some applications, including water-in-oil droplet generation. From Su’s work, the chips that show stable monodisperse droplet generation have a WCA of ∼115°.^16^ In this study, the WCA of the native COC substrate is ∼86°, as shown in Figure 3. The COC substrate coated 20 times with 40% cyclohexane and 1% FOTS show the highest WCA of ∼116.08° (shown in Figure 4c), and its WCA remains unchanged after 3 weeks, which proves the effectiveness and stability of hydrophobic treatment.

The COC substrates treated with 20% cyclohexane and 1% FOTS show the WCA of ∼93.12 to ∼95.17° in Figure 4a, which increases slightly compared with the native COC surface (86°). The coating times and the time after the hydrophobic surface treatment reveal little significance in changing the WCA of the COC surface. The possible reason is that 20% cyclohexane is insufficient to make COC polymer chains fully mobile, thus causing fewer mobile chains to entangle with the FOTS molecules. Generally speaking, the more coating times represent more FOTS modifiers sprayed onto the COC substrate, indicating a higher amount of FOTS molecules spread onto the COC substrate. However, the low concentration of cyclohexane disables the COC polymer chains to move. Under this condition, the coating times and the time after treatment have little effect on the WCA.

For the treatment of COC substrate with 30% cyclohexane and 1% FOTS, all samples show a higher WCA value compared with the samples treated with 20% cyclohexane and 1% FOTS (shown in Figure 4b). This result proves the effectiveness of 30% cyclohexane in dissolving the COC polymer surface and causing polymer chains to be mobile and thus entangled with the FOTS molecules. The WCA increases from 86° before treatment to ∼103.15° for 5 times coating, ∼106.36° for 10 times coating, and ∼105.19° for 20 times coating at 10 min after the surface treatment. For 5 times coating, the WCA increases to ∼105.13° after 2 days of storage and remains almost unchanged (∼106.75°) during 3 weeks of storage. For 10 times coating, the WCA of the COC surface remains ∼106.85° after 2 days of storage and keeps increasing to ∼112.26° at 3 weeks after the treatment. For 20 times coating, the WCA increases to ∼108.85° at 2 days and ∼112.9° at 3 weeks after the treatment. The increase of WCA indicates that the hydrophobic surface is developed by both molecular entanglement between the COC polymer chains and the FOTS molecules, with the assistance of cyclohexane, and by the formation of a Si–O–Si crosslinked network after 2-day or even longer-time storage.^16^

The COC substrate treated with 40% cyclohexane and 1% FOTS shows the highest WCA value after 1 week of storage, with ∼111.49° for 5 times coating, ∼114.00° for 10 times coating, and ∼115.76° for 20 times coating (shown in Figure 4c). And the WCA remains stable after 3 weeks of storage, with ∼111.81° for 5 times coating, ∼115.37° for 10 times coating, and ∼116.08° for 20 times coating. This result proves the effectiveness of a high concentration of cyclohexane in causing COC polymer chains to be more mobile and thereafter entangled with FOTS molecules. The coating times 10 and 20 ensure a sufficient amount of FOTS species embedded onto the COC surface. All treated COC substrates show a delay in surface hydrophobicity, represented as the WCA continuously increasing after the treatment and stabilizing after 1 week. The delay of the hydrophobicity effect proves the two-step mechanism of hydrophobic surface formation, which is molecular entanglement followed by Si–O–Si network formation. To conclude, the COC surface coated with 40% cyclohexane and 1% FOTS for 10 and 20 times has a WCA higher than 115°, which is potential for hydrophobic microfluidics applications, especially for water-in-oil droplet generation.

#### 3.2.2 FTIR Analysis

**Figure 5:**
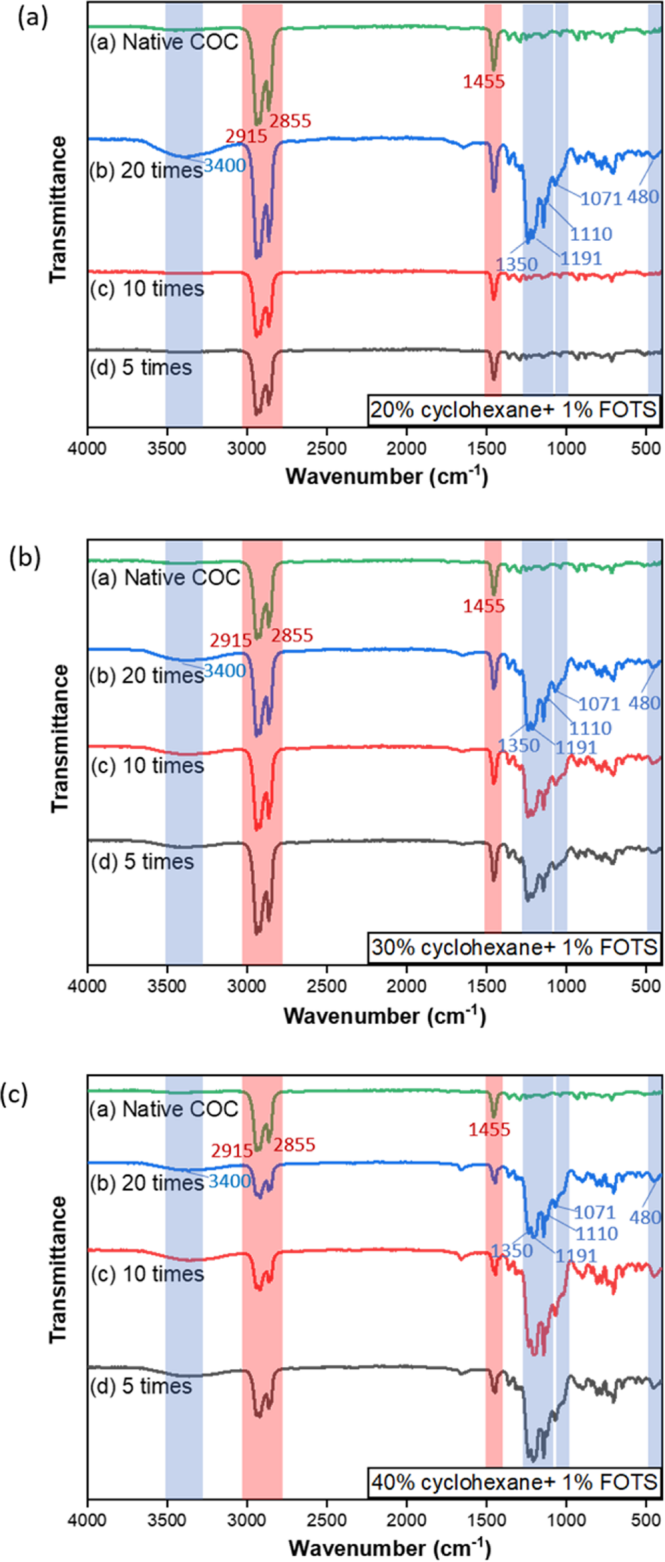
FTIR spectra of the COC substrates treated with (a) 20%, (b) 30%, and (c) 40% cyclohexane and 1% FOTS with different coating times.

The ATR-FTIR analysis was used to identify the chemical bonds on the native COC surface and the change of chemical bonds on the modified surfaces. Figure 5 shows the FTIR spectra of different COC substrates. For the native COC substrate, the bands of (2855 and 2915 cm^–1^) represent the stretching vibration modes of CH_2_ and CH_3_, and the band of (1453 cm^–1^) is attributed to the CH_3_ wagging mode of the polymer backbone (illustrated by the green line in Figure 5a).

For the surfaces treated with 20% cyclohexane and 1% FOTS for 20 times coating, the characteristic absorption bands are identified as Si–O–Si stretching vibration (1071 cm^–1^) and O–Si–O bending vibration (480 cm^–1^) (illustrated by the blue line in Figure 5a), which have also been proved in others’ work.^16^ Meanwhile, the absorption bands can be identified as the CF_2_ group (1191 cm^–1^) and CF_3_ group (1110 and 1350 cm^–1^). The presence of Si–OH is detected at the band (3400 cm^–1^), which occurs during the crosslinking of silane. However, the surfaces treated with 20% cyclohexane and 1% FOTS for 5 times and 10 times coating have the same spectrum as the native COC substrate (illustrated by the black and red lines in Figure 5a). It could be explained that a lower concentration of cyclohexane (20%) with fewer coating times is not sufficient to dissolve the COC surface and form the entrapment layer. Therefore, the COC substrate barely interacts with the low concentration of cyclohexane at low coating times.

The spectra of the COC surfaces treated with 30% cyclohexane and 1% FOTS have the features of both COC and FOTS crosslinked networks since 5 times coating (illustrated by the black line in Figure 5b). Similar spectra features have also been found in the COC substrates coated 10 and 20 times (illustrated by the red and blue lines in Figure 5b), which suggests this concentration is sufficient to dissolve the COC surface and form the entrapment layer since 5 times coating.

Similarly, the COC surfaces treated with 40% cyclohexane and 1% FOTS for 5, 10, and 20 times show the spectra with the features of both COC and FOTS crosslinked networks (illustrated by the black, red, and blue lines in Figure 5c). Therefore, the above chemical bond analysis could verify the effectiveness of the hydrophobic surface treatment. The time delay of the hydrophobic effect could be explained by the formation of Si–OH, which is the result of the interaction between FOTS and water. The Si–OH molecules could further trap FOTS on the COC surface and cause the CF groups to modify the surface chemistry. Similar results have been found in other work, which proved that the fluorinated silane changed the wetting ability of the polymer surface by lowering its surface energy.^21^ The FTIR result also confirms that the entrapment of FOTS occurs only with a sufficient concentration of cyclohexane, which is capable of dissolving the COC surface and causing COC polymer chains to become mobile and entangled with FOTS molecules. This is the reason why almost no difference could be observed for the surfaces treated with 20% cyclohexane and 1% FOTS for 5 and 10 times coating.

From water contact angle measurement and FTIR analysis, it could be concluded that the COC substrates treated with 40% cyclohexane and 1% FOTS 10 and 20 times successfully generate hydrophobic surfaces with the WCA of ∼115°, which could be used for water-in-oil droplet generation, as also proved in Su’s work: the stable monodisperse droplets were generated in the PC chip with the WCA of ∼115°.^16^ In Liu’s work, the water-in-oil droplets could be formed in the polydimethylsiloxane (PDMS) microfluidic chip with a WCA of ∼112°.^22^ Therefore, chips treated under this condition were selected for other characterizations.

#### 3.2.3 Surface Roughness Measurement

**Figure 6:**
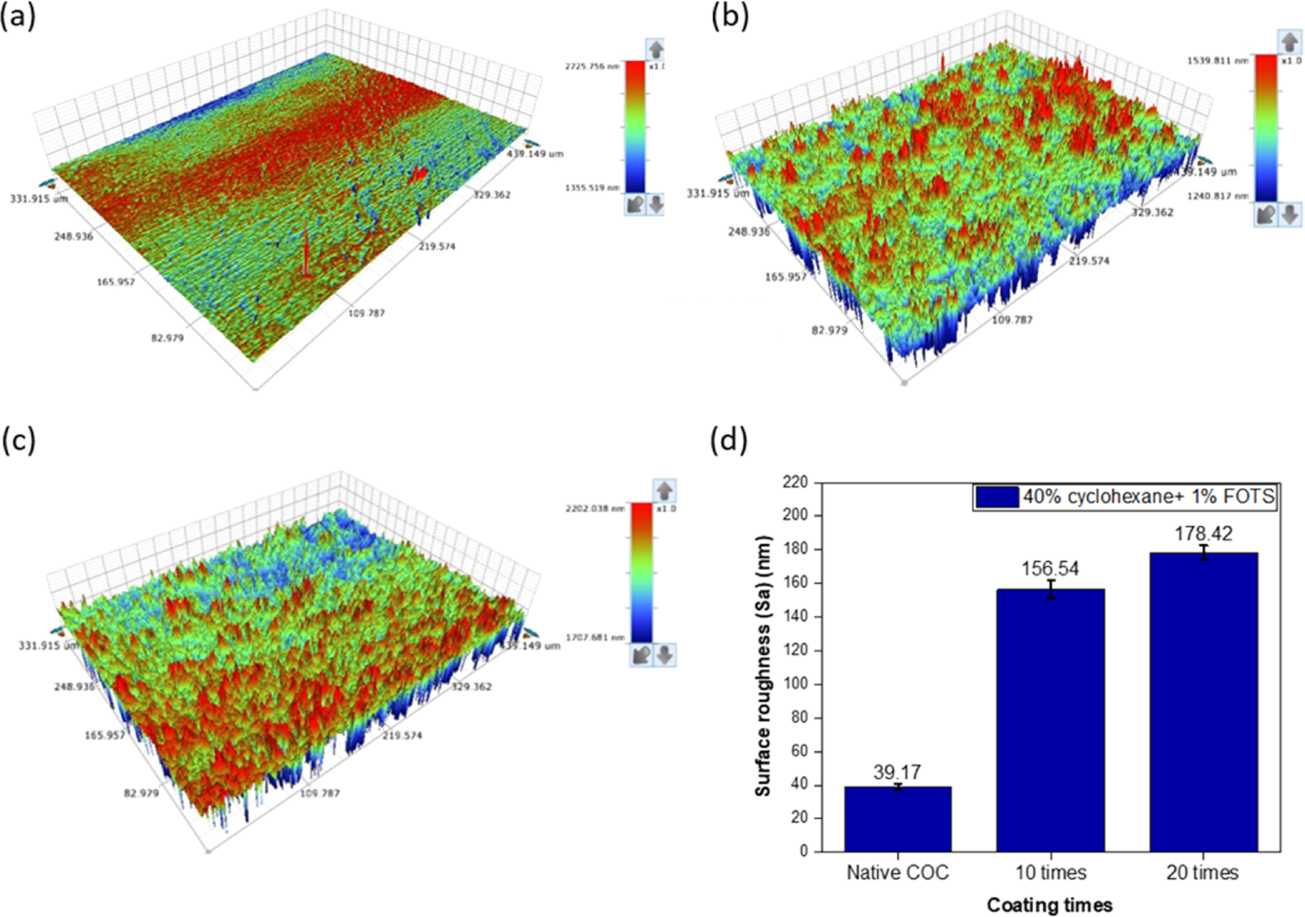
3D images of the surface roughness for COC chips from the profilometer: (a) native COC, (b) COC substrate coated by 40% cyclohexane and 1% FOTS for 10 times, and (c) COC substrate coated by 40% cyclohexane and 1% FOTS for 20 times. (d) Surface roughness value of native COC substrate and COC substrates coated by 40% cyclohexane and 1% FOTS for different times.

The COC substrates treated with 40% cyclohexane and 1% FOTS for 10 and 20 times were characterized by a profilometer to detect the effect of surface treatment on the surface roughness (*S*_a_) of the substrate. The *S*_a_ value is ∼39.17 nm for the untreated COC surface. After hydrophobic treatment, *S*_a_ increases to ∼156.54 and ∼178.42 nm for the substrate coated 10 and 20 times, respectively (Figure 6d). The exposure to 40% cyclohexane roughens the COC surface, causing its surface to swell and form a sticky bonding layer. It is possible that each time the cyclohexane was applied, the COC surface was dissolved layer by layer, which may lead to uneven texture when the surface was dried. Therefore, the more coating times were applied, the thicker sticky bonding layer was developed,^16^ resulting in a higher *S*_a_ value. More importantly, it could be observed from the 3D image that the surface texture is uniform due to the application of ultrasonic spray coating, which confirms the effectiveness of ultrasonic spray coating.

#### 3.2.4 Optical Clarity Characterization

**Figure 7:**
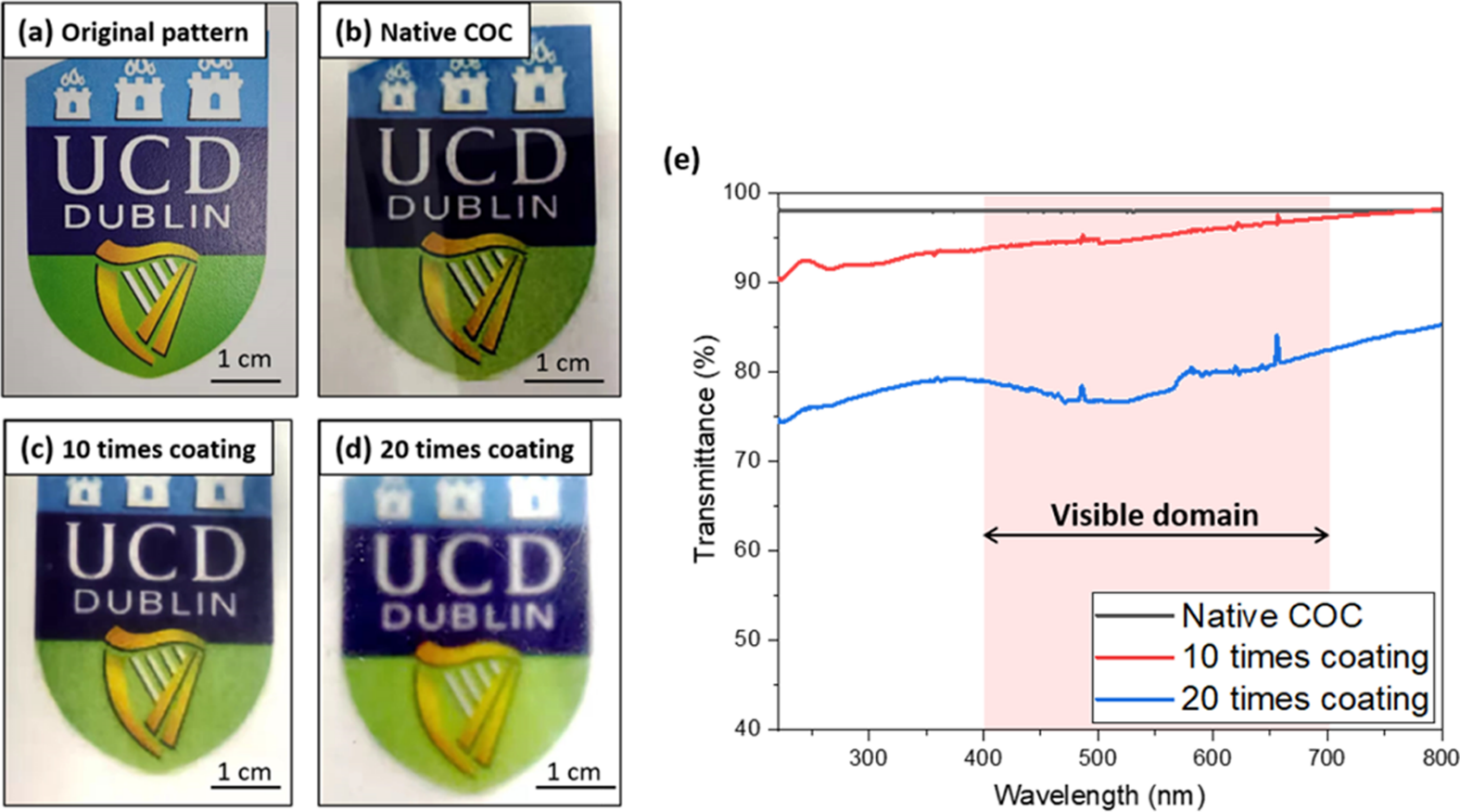
Influence of the coating times on the optical clarity of the COC films: (a) Original pattern; (b) native COC film; (c) COC film coated with 40% cyclohexane and 1% FOTS for 10 times; (d) COC film coated with 40% cyclohexane and 1% FOTS for 20 times; and (e) UV–vis transmittance spectra of native COC film and COC film treated with 10 and 20 times coating.

As shown in Figure 7, the effect of coating times on the optical clarity of the COC film was investigated. The pattern covered by native COC film shows a distinct border (shown in Figure 7b), representing the good optical charity of native COC film. The optical charity of COC film can be maintained by the proper surface treatment with 40% cyclohexane and 1% FOTS for 10 times coating. The border of the pattern gradually becomes indistinct with the increase of the coating times (shown in Figure 7d), indicating that the COC film coated with 40% cyclohexane and 1% FOTS for 20 times have lower optical clarity compared with the film coated for 10 times. Figure 7d shows the UV–vis optical transmittance of native COC film, and the COC film treated with different coating times. Compared with native COC film, the transmittance decreased slightly (0–8%) for 10-times-coated film. While for 20-times-coated film, the decrease of transmittance is more significant (13–23%). The optical clarity change is possibly due to the increment of the surface roughness, as it has been proved in others’ work that the change of surface roughness directly influences the transparency of the polymer microfluidic devices.^23^ It should be noted that, the 10-times-coated COC film has the transmittance of 93.8–97.5% in the visible domain (400–700 nm), indicating that this coating technique is applicable to the optical COC devices in diverse applications, as also discussed in other study.^24^ Therefore, proper treatment times and concentration optimization is important to attain the required transparency for detection.

In summary, the COC substrates treated with 40% cyclohexane and 1% FOTS 10 and 20 times achieved desired hydrophobicity, which were selected for bonding optimization. In practical applications, coating conditions could be customized according to the desired optical requirements.

### 3.3 Solvent-Assisted Thermal Bonding Optimization

After the COC film and chip were treated with 40% cyclohexane and 1% FOTS 10 and 20 times, they were prepared for bonding. To investigate the effect of solvent exposure time on the bonding effect and bonding strength, we prepared the chips with different exposure time (60 and 120 s) in solvent-assisted thermal bonding, while the composition of solvent was fixed at 60% cyclohexane and 40% acetone and the bonding temperature was set at 72 °C, which is below the *T*_g_ of COC 8007 (78 °C). Our previous study found that when the exposure time was less than 60 s, the film and chip cannot be bonded due to the lack of mobile polymer chains; when the exposure time exceeded 120 s, the channels deformed after chips and films were bonded. Therefore, 60 and 120 s were selected for bonding optimization. Similarly, the bonding temperature was optimized to be 72 °C, as lower temperatures led to insufficient bonding while higher temperatures caused channels’ deformation.

#### 3.3.1 Leakage Test

**Figure 8:**
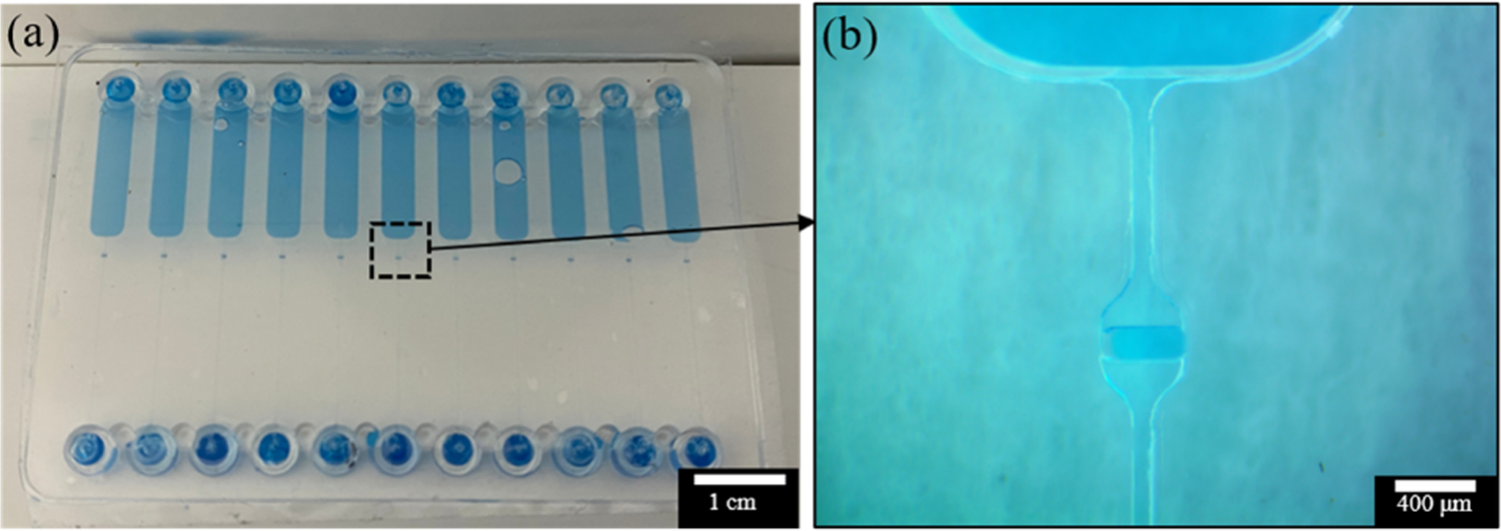
(a) Images of blue dye flowing through the chip channels and (b) the microchannel filled with the blue dye under the optical microscope. The chip was coated with 40% cyclohexane and 1% FOTS 10 times, followed by 120 s solvent exposure during bonding. No leakage was observed at the pressure of ∼500 kPa.

A leakage test was performed by injecting blue-colored water into the chip channel to determine whether the chips could remain functional after the surface treatment and bonding. For untreated chips, all chips were successfully bonded with 60% cyclohexane and 40% acetone, and all channels functioned well without leakage after bonding. For the chips treated with 1% FOTS 10 times, the 60 s exposure achieved a lower bonding strength, as some channels did not function after bonding. However, when the film was exposed to a solvent mixture for 120 s, the chip was successfully bonded and remained functional after bonding (Figure 8a). Figure 8b shows the microchannel remained functional after the hydrophobic treatment and bonding process. Under this condition, the 120 s exposure could cause the COC chains on the upper layer to become mobile and fully entangled with the COC chains on the top surface of the chip substrate during thermal bonding. When the coating times increased to 20 times, some channels lost function after bonding, and leakage was observed for moderate syringe pressure (∼300 kPa). This is because a large amount of FOTS molecules are entrapped on the top layer of the COC film cover and chip, which could increase the hydrophobicity of the interface and potentially reduce the bonding effect, as also proved in other research.^16^

#### 3.3.2 Bonding Strength Characterization

**Figure 9:**
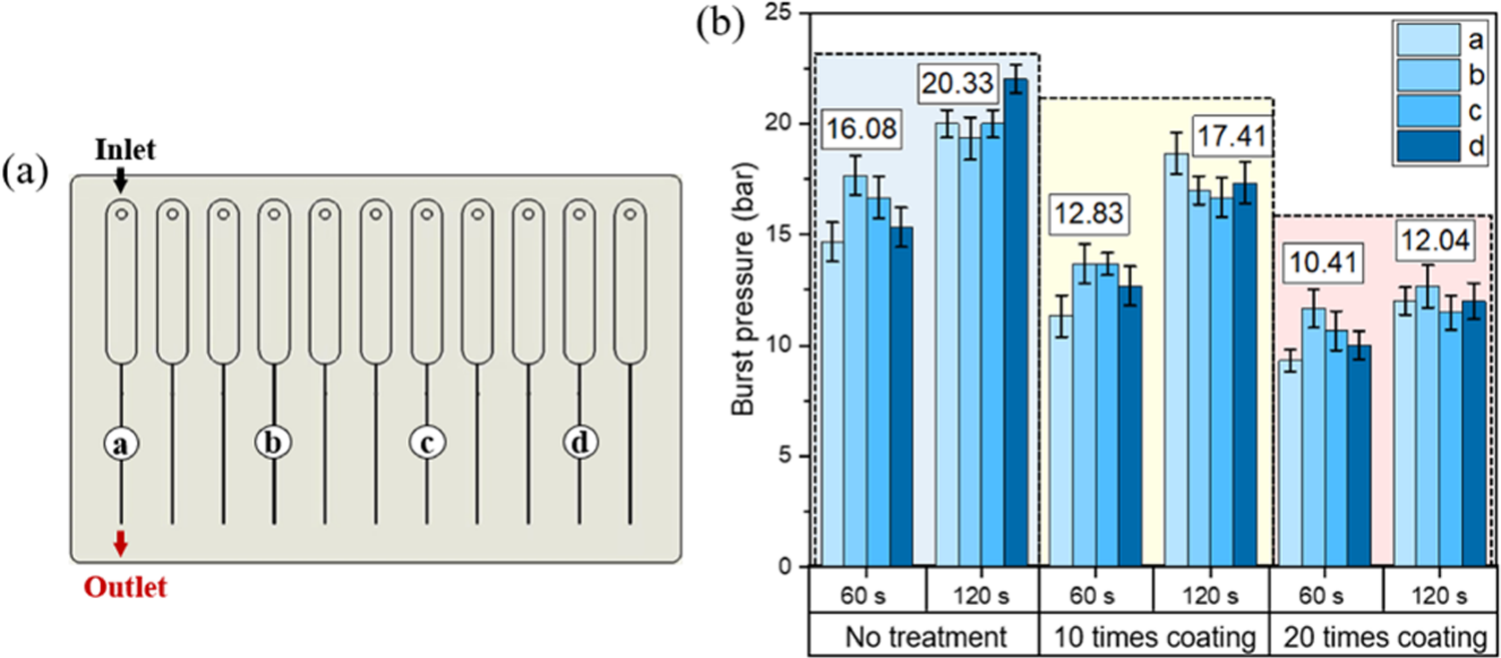
Schematic of four selected channels on the bonded chip (a) and the relationship between different surface treatment conditions and solvent exposure time and the burst pressure of the microfluidic chips (b). The average burst pressure of four channels is labeled at the top of each group of columns.

The bonding strength was evaluated by testing the burst pressure of four different channels in each bonded chip. As shown in Figure 9a, four different channels were selected for bonding strength characterization. Figure 9b shows that the position of the channels on the chip does not affect the bonding strength significantly, indicating that the bonding effect across the whole chip is uniform. Moreover, for both treated and untreated samples, the burst pressure increases with the increase of solvent exposure time. The COC chips with 120 s exposure always have higher burst pressure than those with 60 s exposure under the same surface treatment condition (shown in Figure 9b). This is because more solvent exposure can dissolve more COC chains on the top surface of the film, causing more COC chains to be mobile and diffuse across the surface, thus resulting in a higher bonding strength. Meanwhile, the surface treatment also affects the bonding strength. For the chips exposed to the solvent mixture for 60 s, the burst pressure decreases with the increase of coating times, represented as the average burst pressure decreasing from 16.08 bar for nontreated chips to 12.83 bar for 10-times-treated chips and 10.41 bar for 20-times-treated chips. Similarly, for the chips exposed for 120 s, the average burst pressure decreases from 20.33 bar for nontreated chips to 17.41 bar for 10-times-treated chips and 12.04 bar for 20-times-treated chips. It could be concluded that the hydrophobic treatment reduces the bonding strength. The surface modification proves to fix the FOTS modifier on the surface of the chip and film. FOTS molecules may obstruct the bonding process, which requires exposing cyclohexane to the COC chains and fusing the COC chains between the film cover and the COC chip. Additionally, more coating times will spray too much cyclohexane on the COC surface, resulting in a thicker entrapment layer.^16^ This thick entrapment layer is not as dense as the native COC substrate, reducing the bonding strength between two COC surfaces.

It should be noted that, among all treated chips, the chip that demonstrates the maximum burst pressure (17.41 bar) is treated with 40% cyclohexane and 1% FOTS 10 times, followed by 120 s solvent exposure. This bonding strength is sufficient for droplet generation, as it is higher than the typical pressure required for droplet generation.^25^ Therefore, the ultrasonic spray surface treatment with 40% cyclohexane and 1% FOTS for 10 times coating followed by 120 s solvent exposure was selected as the optimized surface treatment and bonding conditions for subsequent cross-section analysis.

#### 3.3.3 Cross-Section Analysis

**Figure 10:**
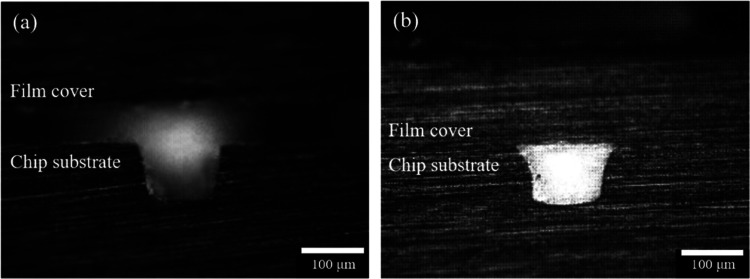
Optical images of microchannels on (a) nonbonded COC chip and (b) COC chip coated with 40% cyclohexane and 1% FOTS 10 times followed by exposure to 60% cyclohexane and 40% acetone for 120 s and then thermal bonded at 72 °C. The bottom width of the channel is ∼100 μm, and the height is ∼100 μm. The channel’s sloped wall results from the draft angle of the micro structures on the stainless-steel mold.

The chips treated with 40% cyclohexane and 1% FOTS for 10 times coating followed by 120 s solvent exposure was selected for cross-section analysis. As shown in Figure 10, after bonding, the coated and bonded chip maintained its integrity compared with the original COC chip. The holding temperature during the thermal bonding is 72 °C, below the *T*_g_ of COC 8007 (78 °C). Therefore, the COC substrates should remain in the solid state without any deformation.

In summary, the optimized treatment and bonding conditions for this study are that the COC chip and film are coated with 40% cyclohexane and 1% FOTS 10 times, then exposed to 60% cyclohexane and 40% acetone for 120 s and then thermal bonded at 72 °C. The chips bonded under this condition show the highest bonding strength while maintaining the channel integrity without collapse.

### 3.4 Characterization of Chips Fabricated by Hydrophobic Treatment and Solvent-Assisted Thermal Bonding

Considering the surface treatment and bonding characterization, the chips treated with 40% cyclohexane and 1% FOTS for 10 and 20 times develop hydrophobic surfaces; the cover films exposed to the solvent mixture for 120 s generate a relatively higher bonding strength after bonding. Therefore, chips and films treated and bonded under these conditions were selected for hydrophobicity characterization.

#### 3.4.1 Water Contact Angle Measurement

After surface treatment and bonding, the water contact angle (WCA) on the excess film cover was measured to evaluate the stability of hydrophobic surface treatment. It is expected that the hydrophobicity was not affected by solvent exposure as well as the heat and pressure applied during solvent-assisted thermal bonding. As shown in Table 2, the chip bonded without surface treatment has the WCA of 86 ± 1.7°, while the chips first treated with 40% cyclohexane and 1% FOTS for 10 and 20 times and then bonded have the WCAs of 115 ± 1.2 and 115 ± 1.8°, respectively. This result proves the effectiveness and stability of hydrophobic surface treatment.

**Table 2:**
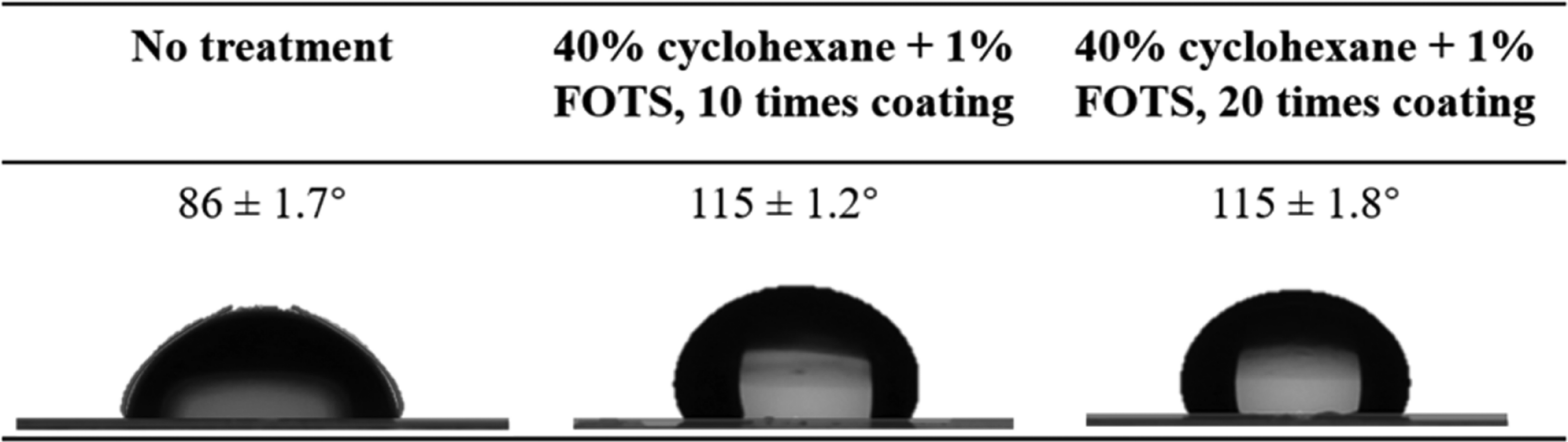
Comparison of Hydrophobicity among the Bonded Native COC Film, COC Film Treated with 40% Cyclohexane and 1% FOTS for 10 and 20 Times and then Bonded^a^

a During the bonding process, the exposure time for all samples was fixed at 120 s.

#### 3.4.2 Capillary Effect Evaluation

**Figure 11:**
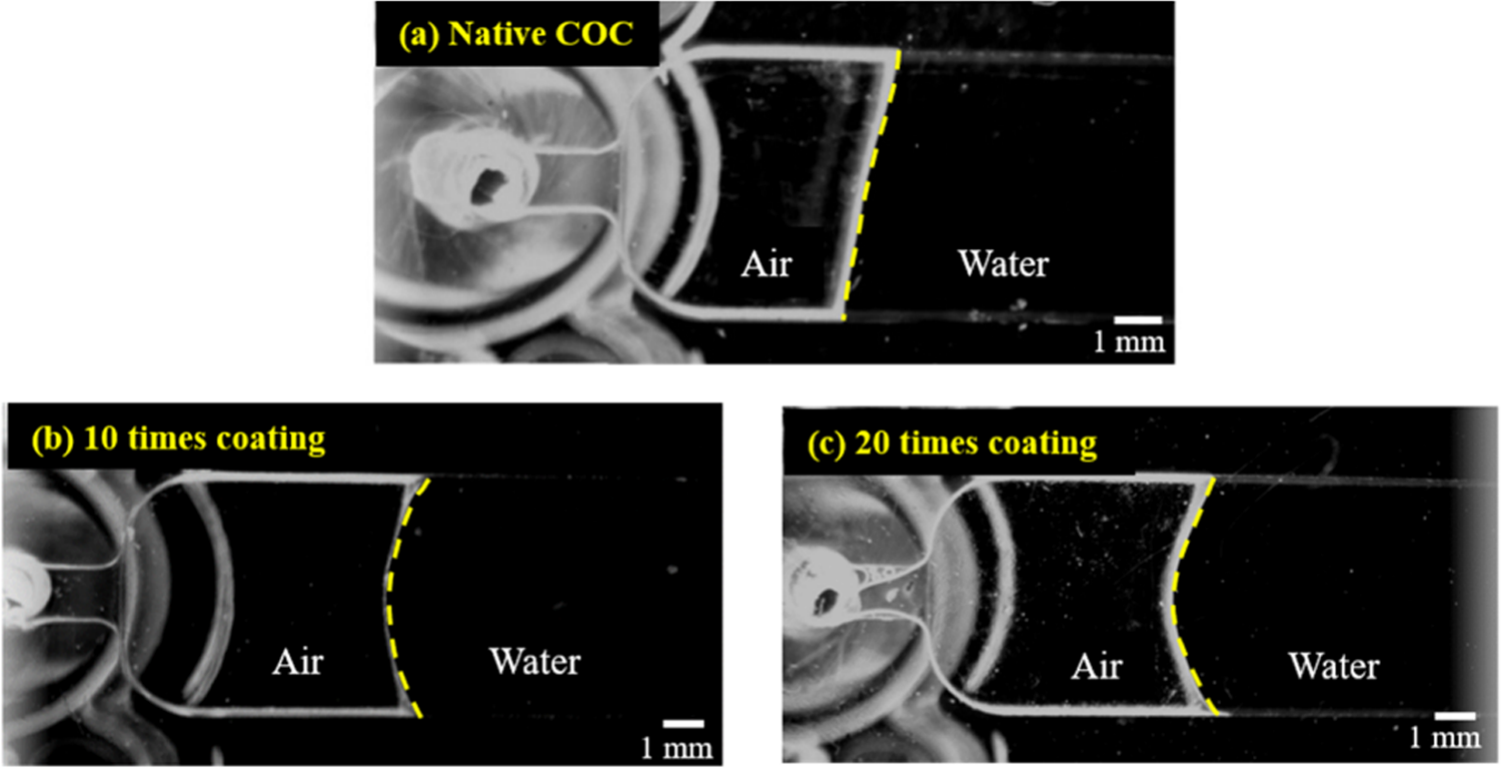
Capillary effect of injected DI water; (a) bonded COC chip without surface treatment, (b) COC chip treated with 40% cyclohexane and 1% FOTS for 10 times and then bonded, and (c) COC chip treated with 40% cyclohexane and 1% FOTS for 20 times and then bonded. During the bonding process, the exposure time for all samples was fixed at 120 s.

To evaluate the hydrophobicity of channels after treatment and bonding, the DI water was injected into the chip channel, and the shape of the water-air interface was observed under a microscope. As shown in Figure 11, the untreated COC chip has a flat flow interface, indicating the wettability of the channel is good. As for the COC chips treated with 40% cyclohexane and 1% FOTS 10 and 20 times and then bonded, the shape of the water flow is more curved due to the lack of wetting in the microchannel surfaces.^26^ Therefore, the surface hydrophobicity inside the channel is well maintained after bonding.

### 3.5 Summary of Bonding Techniques

For the mass production of microfluidic chips, four prevalent bonding techniques are used: laser welding, ultrasonic welding, thermal diffusion bonding, and solvent-assisted thermal bonding.^7,17^Table 3 compares the advantages and limitations of each method. Bonding itself is a challenging task for the fabrication of microfluidic chips. Combining bonding and surface treatment is even more challenging and largely influences the microfluidic cartridge cost since they account most of cycle time of chip production. Hydrophilic treatment such as oxygen plasma and UV/ozone can be conducted before bonding and benefits to increase the wettability of the chips, reducing the surface energy and improving the bonding strength.^12^ However, most bonding techniques are not applicable with hydrophobic surface treatment due to the difficulty of mating surfaces with decreased surface tension after the hydrophobic treatment.^16^ Laser welding and ultrasonic welding require plasma-enhanced chemical vapor deposition (PECVD) as hydrophobic surface treatment to modify the wettability of microchannels in the sealed chip.^27^ However, it is difficult to coat narrow channels and holes with PECVD, and the coatings produced from PECVD may result in a poor uniformity,^28^ due to limited diffusion from bonded channels with small size after bonding. In this study, ultrasonic spray coating is applied to uniformly coat the modifier onto the chip and film surface before bonding, which provides a more effective and less time-consuming hybrid surface hydrophobic treatment and bonding technology. The nanodroplets from the ultrasonic nozzle are capable of covering the small features in the chip, thus ensuring the uniformity of the coating. The solvent-assisted thermal bonding provides the high bonding strength, and the 2-day post-treatment requires no labor work, which enables the cost-effective scale-up of the chip production from injection molding to surface treatment and bonding.

**Table 3:** Comparison of Different Bonding Techniques for the Mass Production of Microfluidic Devices

bonding method	bonding time	bonding strength (represented as burst pressure)	advantages	limitations	applicable surface treatment	references
laser welding	∼10 s	up to 10 bar (medium)	low temperature, pure, and strong bond, localized bonding	can be costly for complex microfluidic structures; need a nontransparent part for bonding	plasma-enhanced chemical vapor deposition (PECVD), bonding is conducted before PECVD, thus difficult to treat small channels after sealing	(29)
ultrasonic welding	several to 30 s	up to 10 bar (medium)	low temperature, localized bonding, rapid welding process, no curing, or solvent involved	not accurate for very small and intricate features; energy director is required	PECVD, difficult to treat small channels after sealing	(30, 31)
thermal diffusion bonding	several to 30 min	5–10 bar (medium)	low cost, simple operation, no adhesive clogging	heating temperature higher than the *T*_g_ of polymer, prone to cause channel distortion	can be treated with UV/ozone or plasma to acquire hydrophilic surfaces, but hard to be combined with hydrophobic treatment	(32, 33)
solvent-assisted thermal bonding	several to 30 min	10–100 bar (high)	simple operation, low temperature, low cost	the process requires optimization to prevent the channel from collapsing due to polymer softening	can be combined with spray coating as the surface treatment, which takes at least 2 days as the post-treatment	(16, 30, 33)
our bonding technique	16 min for solvent-assisted bonding, 2 days for post-treatment (no labor work needed)	∼17 bar (high)	simple operation, low cost, low temperature, large-scale production	the post-treatment for the generation of a stable hydrophobic surface takes ∼2 days	combined with ultrasonic spray coating prior to bonding, ensuring the uniformity of coating and maintaining a high bonding strength	

## 4 Conclusions

In summary, a hydrophobic surface treatment followed by solvent-assisted thermal bonding was successfully developed to modify and bond COC chips for microfluidic applications, including water-in-oil droplet generation. The mechanism for entrapment functionalization and solvent-assisted thermal bonding was analyzed. The COC chips coated with 40% cyclohexane and 1% FOTS by ultrasonic spray 10 and 20 times have a water contact angle of ∼115°, and this hydrophobicity could be maintained after the storage of 3 weeks. The FTIR results confirmed the surface functionalization by revealing the formation of Si–OH and Si–O–Si groups, indicating the crosslinking of silane after the surface treatment. The surface roughness of 10 times coated chips is lower, presenting better optical clarity. After the surface treatment, the solvent-assisted thermal bonding was optimized to yield a bonding strength over 17 bar (represented as the burst pressure of bonded chips), which is sufficient for droplet generation. The COC chip under 120 s exposure to 60% cyclohexane and 40% acetone shows a maximum bonding strength and maintains its channel integrity without any water leakage during the characterization. The chips were uniformly bonded, represented as the similar burst pressure of different channels on the chip. The hydrophobicity was maintained after the bonding process, with the water contact angle of ∼115° on the film cover and the curved shape of water flow inside the channel, which is due to the lower wettability of the channel wall after bonding.

This work provides a repeatable and scalable hydrophobic surface modification process, which entraps FOTS molecules on the polymer surface through ultrasonic spray coating. Meanwhile, a solvent vapor-assisted thermal bonding method is developed in a controllable way to achieve a high bonding strength and meanwhile maintain the channel integrity. Compared to other assembly processes requiring hydrophobic surface modification, these two methods can be combined sequentially for large-scale production of plastic microfluidic cartridges with shorter fabrication time and lower cost. If extended, such a process also has a high potential for microfluidic applications with hydrophilic coatings.

## References

[ref1] Ayala R.; Zhang C.; Yang D.; Hwang Y.; Aung A.; Shroff S. S.; Arce F. T.; Lal R.; Arya G.; Varghese S. Engineering the cell–material interface for controlling stem cell adhesion, migration, and differentiation. Biomaterials 2011, 32, 3700–3711. 10.1016/j.biomaterials.2011.02.004. 21396708

[ref2] Suzuki K. Flow resistance of a liquid droplet confined between two hydrophobic surfaces. Microsyst. Technol. 2005, 11, 1107–1114. 10.1007/s00542-005-0510-z.

[ref3] Shang L.; Cheng Y.; Zhao Y. Emerging droplet microfluidics. Chem. Rev. 2017, 117, 7964–8040. 10.1021/acs.chemrev.6b00848. 28537383

[ref4] Teh S.-Y.; Lin R.; Hung L.-H.; Lee A. P. Droplet microfluidics. Lab Chip 2008, 8, 198–220. 10.1039/b715524g. 18231657

[ref5] Zhang N.; Srivastava A.; Kirwan B.; Byrne R.; Fang F.; Browne D. J.; Gilchrist M. D. Manufacturing microstructured tool inserts for the production of polymeric microfluidic devices. J. Micromech. Microeng. 2015, 25, 095005 10.1088/0960-1317/25/9/095005.

[ref6] Perez-Toralla K.; Champ J.; Mohamadi M. R.; Braun O.; Malaquin L.; Viovy J.-L.; Descroix S. New non-covalent strategies for stable surface treatment of thermoplastic chips. Lab Chip 2013, 13, 4409–4418. 10.1039/c3lc50888a. 24061577

[ref7] Tsao C.-W.; DeVoe D. L. Bonding of thermoplastic polymer microfluidics. Microfluid. Nanofluid. 2009, 6, 1–16. 10.1007/s10404-008-0361-x.

[ref8] Zhang Y.; Gao K.; Fan Y. Application of a new UV curable adhesive for rapid bonding in thermoplastic-based microfluidics. Micro Nano Lett. 2019, 14, 211–214. 10.1049/mnl.2018.5479.

[ref9] Sun Y.; Kwok Y. C.; Nguyen N.-T. Low-pressure, high-temperature thermal bonding of polymeric microfluidic devices and their applications for electrophoretic separation. J. Micromech. Microeng. 2006, 16, 1681 10.1088/0960-1317/16/8/033.

[ref10] Uba F. I.; Hu B.; Weerakoon-Ratnayake K.; Oliver-Calixte N.; Soper S. A. High process yield rates of thermoplastic nanofluidic devices using a hybrid thermal assembly technique. Lab Chip 2015, 15, 1038–1049. 10.1039/C4LC01254B. 25511610 PMC4315742

[ref11] Ogilvie I.; Sieben V.; Floquet C.; Zmijan R.; Mowlem M.; Morgan H. In Solvent Processing of PMMA and COC Chips for Bonding Devices with Optical Quality Surfaces, 14th international conference on miniaturized systems for chemistry and life sciences, 2010; pp 1244–1246.

[ref12] Tsao C. W.; Hromada L.; Liu J.; Kumar P.; DeVoe D. Low temperature bonding of PMMA and COC microfluidic substrates using UV/ozone surface treatment. Lab Chip 2007, 7, 499–505. 10.1039/b618901f. 17389967

[ref13] Ghosh S.; Kamalakshakurup G.; Lee A. P.; Ahn C. H. A mass manufacturable thermoplastic based microfluidic droplet generator on cyclic olefin copolymer. J. Micromech. Microeng. 2019, 29, 055009 10.1088/1361-6439/ab0e60.

[ref14] Sarmadi M. In Advantages and Disadvantages of Plasma Treatment of Textile Materials, 21st International Symposium on Plasma Chemistry (ISPC 21), Sunday, 2013.

[ref15] Ruckenstein E.; Li Z. Surface modification and functionalization through the self-assembled monolayer and graft polymerization. Adv. Colloid Interface Sci. 2005, 113, 43–63. 10.1016/j.cis.2004.07.009. 15763238

[ref16] Su S.; Jing G.; Zhang M.; Liu B.; Zhu X.; Wang B.; Fu M.; Zhu L.; Cheng J.; Guo Y. One-step bonding and hydrophobic surface modification method for rapid fabrication of polycarbonate-based droplet microfluidic chips. Sens. Actuators, B 2019, 282, 60–68. 10.1016/j.snb.2018.11.035.

[ref17] Keller N.; Nargang T. M.; Runck M.; Kotz F.; Striegel A.; Sachsenheimer K.; Klemm D.; Länge K.; Worgull M.; Richter C.; et al. Tacky cyclic olefin copolymer: a biocompatible bonding technique for the fabrication of microfluidic channels in COC. Lab Chip 2016, 16, 1561–1564. 10.1039/C5LC01498K. 27040493

[ref18] Guo H.; Ulbricht M. The effects of (macro) molecular structure on hydrophilic surface modification of polypropylene membranes via entrapment. J. Colloid Interface Sci. 2010, 350, 99–109. 10.1016/j.jcis.2010.06.032. 20621303

[ref19] Badv M.; Jaffer I. H.; Weitz J. I.; Didar T. F. An omniphobic lubricant-infused coating produced by chemical vapor deposition of hydrophobic organosilanes attenuates clotting on catheter surfaces. Sci. Rep. 2017, 7, 11639 10.1038/s41598-017-12149-1. 28912558 PMC5599680

[ref20] Matsumoto N. Overview of silicon-based materials. Jpn. J. Appl. Phys. 1998, 37, 5425–5436. 10.1143/JJAP.37.5425.

[ref21] Subramanian B.; Kim N.; Lee W.; Spivak D. A.; Nikitopoulos D. E.; McCarley R. L.; Soper S. A. Surface modification of droplet polymeric microfluidic devices for the stable and continuous generation of aqueous droplets. Langmuir 2011, 27, 7949–7957. 10.1021/la200298n. 21608975 PMC3443641

[ref22] Liu F.; Xu T.; Liu W.; Zheng X.; Xu J.; Ma B. Spontaneous droplet generation via surface wetting. Lab Chip 2020, 20, 3544–3551. 10.1039/D0LC00641F. 32895671

[ref23] Roy E.; Pallandre A.; Zribi B.; Horny M. C.; Delapierre F. D.; Cattoni A.; Gamby J.; Haghiri-Gosnet A. M. Overview of materials for microfluidic applications. Adv. Microfluid.: New Appl. Biol., Energy, Mater. Sci. 2016.

[ref24] El Fissi L.; Vandormael D.; Houssiau L.; Francis L. A. Surface functionalization of cyclic olefin copolymer (COC) with evaporated TiO2 thin film. Appl. Surf. Sci. 2016, 363, 670–675. 10.1016/j.apsusc.2015.11.234.

[ref25] Schuler F.; Trotter M.; Geltman M.; Schwemmer F.; Wadle S.; Domínguez-Garrido E.; López M.; Cervera-Acedo C.; Santibáñez P.; von Stetten F.; et al. Digital droplet PCR on disk. Lab Chip 2016, 16, 208–216. 10.1039/C5LC01068C. 26610263

[ref26] Long J.; Weng Q.; Hong W.; Cao Z.; Zhou P.; Xie X. Fast water flow in laser micromachined microgrooves with nonuniform surface wettability. Exp. Therm. Fluid Sci. 2019, 103, 9–17. 10.1016/j.expthermflusci.2018.12.031.

[ref27] aLee K. S.; Ram R. J. Plastic–PDMS bonding for high pressure hydrolytically stable active microfluidics. Lab Chip 2009, 9, 1618–1624. 10.1039/b820924c. 19458871

[ref28] Hosseini S.; Ibrahim F.; Djordjevic I.; Koole L. H. Recent advances in surface functionalization techniques on polymethacrylate materials for optical biosensor applications. Analyst 2014, 139, 2933–2943. 10.1039/c3an01789c. 24769607

[ref29] aDe Pelsmaeker J.; Graulus G.-J.; Van Vlierberghe S.; Thienpont H.; Van Hemelrijck D.; Dubruel P.; Ottevaere H. Clear to clear laser welding for joining thermoplastic polymers: A comparative study based on physicochemical characterization. J. Mater. Process. Technol. 2018, 255, 808–815. 10.1016/j.jmatprotec.2017.12.011.

[ref30] Ereku L.; Mackay R.; Balachandran W.; Ajayi K. Review of commercially available microfluidic materials and fabricating techniques for point of care testing. Sens. Transducers 2016, 202, 1–24.

[ref31] Zhang Z.; Luo Y.; Wang X.; Zheng Y.; Zhang Y.; Wang L. A low temperature ultrasonic bonding method for PMMA microfluidic chips. Microsyst. Technol. 2010, 16, 533–541. 10.1007/s00542-010-1027-7.

[ref32] Jena R.; Chester S.; Srivastava V.; Yue C.; Anand L.; Lam Y. Large-strain thermo-mechanical behavior of cyclic olefin copolymers: application to hot embossing and thermal bonding for the fabrication of microfluidic devices. Sens. Actuators, B 2011, 155, 93–105. 10.1016/j.snb.2010.11.031.

[ref33] Sivakumar R.; Lee N. Y. Microfluidic device fabrication mediated by surface chemical bonding. Analyst 2020, 145, 4096–4110. 10.1039/D0AN00614A. 32451519

